# Dysregulated Hypothalamic–Pituitary–Adrenal Axis Function Contributes to Altered Endocrine and Neurobehavioral Responses to Acute Stress

**DOI:** 10.3389/fpsyt.2015.00031

**Published:** 2015-03-13

**Authors:** Scott A. Kinlein, Christopher D. Wilson, Ilia N. Karatsoreos

**Affiliations:** ^1^Department of Integrative Physiology and Neuroscience, Washington State University, Pullman, WA, USA

**Keywords:** corticosterone, allostatic load, prefrontal cortex, hippocampus, *c-Fos*

## Abstract

Organisms react to environmental challenges by activating a coordinated set of brain–body responses known as the stress response. These physiological and behavioral countermeasures are, in large part, regulated by the neuroendocrine hypothalamic–pituitary–adrenal (HPA) axis. Normal functioning of the HPA axis ensures that an organism responds appropriately to altered environmental demands, representing an essential system to promote survival. Over the past several decades, increasing evidence supports the hypothesis that disruption of the HPA axis can lead to dysregulated stress response phenotypes, exacting a physiological cost on the organism commonly referred to as allostatic load. Furthermore, it has been recognized that high allostatic load can contribute to increased vulnerability of the organism to further challenges. This observation leads to the notion that disrupted HPA function and resulting inappropriate responses to stressors may underlie many neuropsychiatric disorders, including depression and anxiety. In the present set of studies, we investigate the role of both the normally functioning and disrupted HPA axis in the endocrine, neural, and behavioral responses to acute stress. Using a model of non-invasive chronic corticosterone treatment in mice, we show that dysregulating the normal function of the HPA leads to a mismatch between the hormonal and neural response to acute stress, resulting in abnormal behavioral coping strategies. We believe this model can be leveraged to tease apart the mechanisms by which altered HPA function contributes to neurobehavioral dysregulation in response to acute stress.

## Introduction

The term stress carries with it negative connotations, but it describes an important neurobehavioral and physiological response that is essential for survival. In response to an environmental stressor, the body quickly orchestrates changes in brain activity followed shortly thereafter by secretion of “stress mediators,” including cytokines, metabolic hormones, and corticosteroids (1–4). These circulating chemical messages then act at peripheral tissues and in the brain to modify physiological processes and behavioral outputs (1, 4). The primary neuroendocrine axis that regulates the stress response in mammals is the hypothalamic–pituitary–adrenal (HPA) axis. Activation of this system is initiated by projections from the brainstem and limbic system to the paraventricular nucleus (PVN) of the hypothalamus (2–4). Upon activation, corticotropin-releasing hormone (CRH) cells in the PVN secrete CRH onto proopiomelanocortin (POMC)-containing cells at the median eminence, triggering release of adrenocorticotropic hormone (ACTH) into the circulation via activation of CRH receptors (2–6). As circulating ACTH levels increase, melanocortin-2 receptors in the adrenal glands are activated, leading to secretion of glucocorticoid hormones (7), primarily cortisol in humans and corticosterone (CORT) in rodents. Circulating CORT causes increased metabolic activity in peripheral tissues and acts centrally at the brain via glucocorticoid receptors (GRs) in order to induce negative feedback in the circuit (1, 8). This negative feedback largely occurs via indirect connections to the hypothalamus [through the bed nucleus of the stria terminalis (BNST) as well as through changes in endocannabinoid signaling in limbic, cortical, and subcortical brain regions] (1, 4, 9, 10). It is well known that two main CORT responsive brain regions – the medial prefrontal cortex (mPFC) and hippocampal formation – act both directly and indirectly to inhibit the drive to CORT production at the level of the amygdala and the PVN (1–4), thus closing the loop and terminating the acute stress response.

The stress response orchestrated by the HPA axis is well-choreographed multi-system response, involving behavioral, physiological, and metabolic responses, with tightly regulated components that need to become active at the appropriate times and in the appropriate contexts (11, 12). Thus, it is not surprising that disruptions in the biological response to stress can lead to changes in neuronal function, behavior, and immune function (13–18). Disorders related to disrupted HPA function include depression, post-traumatic stress disorder (PTSD), metabolic dysfunction, and anxiety disorders (8, 19–22). These disorders affect millions worldwide, inflicting significant economic and personal costs. Developing animal models to understand how dysfunctions in stress responses lead to altered behavioral outputs is an important goal for modern neuroscience, and could lead to direct translational benefits once mechanisms have been elucidated. Thus, determining common themes in stress-related disorders will be a useful approach in isolating relevant factors for further mechanistic investigation.

Since many neuropsychiatric disorders are associated with changes in basal HPA function, as well as with altered responses to stressors, identifying the hormonal and neurobehavioral systems that are affected could provide important insights as to potential mechanisms by which these changes occur. Using low-dose CORT administered in the drinking water of adult mice, our goal was to determine how this treatment affects endocrine, neural, and behavioral responses to acute stress. Our results show that this non-invasive disruption of the HPA can fundamentally alter both neural and behavioral responses to acute stress and demonstrate that this can be a viable model for dissecting the mechanisms by which HPA dysregulation results in inappropriate neurobehavioral responses.

## Materials and Methods

### Animals and corticosterone treatment

Adult male C57/BL6 mice (aged 35–42 days) were obtained from Harlan Laboratories, Inc. Animals were allowed 1 week acclimatization time upon arrival prior to treatment. Animals were then treated with vehicle (1% ethanol) or CORT (Sigma Aldrich, St. Louis, MO, USA; 25 μg/ml in 1% EtOH) for 28 days prior to testing or tissue collection (23–25). Animal weights were measured weekly and solutions were replaced during that time. All animal experiments were conducted with approval of the Washington State Institutional Animal Care and Use Committee.

### Forced-swim test

Acute stress was accomplished using the forced-swim test (FST). Animals were subjected to 10-min FST (4 l glass beaker, water temperature of 21–23°C) at ZT6. Following FST, animals were allowed to recover for 10 min in their home cage prior to the open-field test (OFT), blood collection, or RT-PCR. Time spent struggling (defined as limb movements in excess of those needed to simply stay afloat) or time spent immobile was scored by an experimenter blinded to the conditions. Swim chambers were emptied, cleaned, and refilled between each trial.

### Corticosterone and adrenocorticotropic hormone RIA

To determine changes in diurnal plasma CORT following vehicle or chronic CORT treatment, a group of mice (*N* = 4–5/group/time point) was killed by rapid decapitation at 6 h intervals at zeitgeber times 0, 6, 12, and 18 (with ZT0 being time of lights ON, and ZT12 being time of lights OFF). To determine endocrine stress responses, a separate group of mice was killed 10 min after the 10-min FST by rapid decapitation (*N* = 4–5/group/treatment). In both cases, trunk blood was collected in EDTA-coated tubes. Brains from the FST group were then used to detect stress-induced mRNA changes via RT-PCR (see below). Blood was spun at 1500 rcf for 15 min in a refrigerated centrifuge, and plasma removed, aliquoted, and stored at −80°C until assay. Plasma was assayed for total CORT using the Siemens Coat-a-Count kit for rat CORT according to the manufacturer’s protocol. To further probe the endocrine response, plasma ACTH was measured in a previously unthawed plasma aliquot from a subset of stressed vehicle- or CORT-treated mice (*N* = 5/group), with an ACTH 125I Kit (DiaSorin, Inc., Stillwater, MN, USA) using the option A (overnight incubation) protocol. For both assays, tubes were counted on a Packard Cobra II gamma counter, and the intrassay coefficient of variability was less than 10%.

### Novel open-field test

To test behavioral responses following FST, an OFT was conducted. The OFT consisted of a white plastic chamber (27.5 cm × 27.5 cm × 20 cm). Lighting was set at 13–18 lux at floor level. Ten minutes following the end of FST (or at ZT6 for unstressed controls, *N* = 8/group/treatment), each animal was placed in the corner of the chamber and allowed to roam freely for 5 min before being removed. The chamber was cleaned with 70% ethanol after each trial. Locomotor behavior was scored digitally using Noldus Ethovision XT software (Leesburg, VA, USA). Animal location was determined by the center point of the animal.

### *In situ* hybridization film autoradiography

To detect CRH mRNA, *in situ* hybridization and film autoradiography was used as previously described (26), with a mouse CRH riboprobe generously provided by Dr. A. Jasnow, Kent State University, OH, USA (27). Briefly, a separate group of unstressed vehicle- or CORT-treated mice (25 μg/ml; *N* = 4–5/group) was killed via rapid decapitation at ZT6, and brains were removed and immediately frozen on dry ice. Brain sections were made on a cryostat (20 μm thick) and mounted on slides. Slide-mounted sections were fixed in 3.7% formaldehyde for 10 min, rinsed in a series of phosphate-buffered saline (PBS) washes, followed by rinses in triethanolamine-HCl (TEA) and TEA with acetic anhydride. Slides were then washed in 2× sodium chloride citrate (SCC) and dehydrated in a series of graded alcohols (70, 95, and 100%), delipidated in chloroform, and rinsed in 100% alcohol. For pre-hybridization, slides were exposed to hybridization solution (225 μl/slide), coverslipped, placed in a humidity chamber, and incubated at 55°C for 1 h. Slides were then washed in 2× SCC and again dehydrated in a series of graded alcohols (70 and 95%). For hybridization, slides were exposed to hybridization buffer (225 μl/slide) with the 35S-labeled antisense or sense ribonucleotide probes (approximately 1 × 10^6^ cpm/slide), coverslipped, placed in a humidity chamber, and incubated at 55°C overnight. Following hybridization, slides were washed in 2× SCC buffer and incubated with RNase A (10 μg/ml) in digestion buffer at 37°C for 30 min and then digestion buffer alone for 10 min. Slides were then rinsed in series of 2× SCC and 0.2× SCC washes at 55°C and dehydrated in a series of graded alcohols. Slides were air dried for 24 h and then apposed to Kodak BioMax MR film (Sigma) for 2 days to generate autoradiograms.

### Quantification of CRH mRNA

Relative optical densities (RODs) were measured from the autoradiograms using computerized image analysis software (MCID-M4, Imaging Research, Inc., St. Catharines, Canada). Background measurements were made from areas adjacent to the PVN and subtracted from the ROD. The same size circular tool, smaller than the size of the brain region (PVN or amygdala) being measured, was used to ensure that the same size sample was measured from section to section, and mouse to mouse. At least two bilateral measurements were made for each mouse.

### Quantitative RT-PCR

As above, brains from each group were collected 10 min after the end of a 10-min FST (*N* = 4–5/group/treatment), and frozen immediately on dry ice. Brain regions of interest were punched using 0.5 mm ID biopsy corer (Fine Science Tools, Foster City, CA, USA) and kept at −80°C until extraction. mRNA isolation was performed using Qiazol (Trizol-chloroform) extraction with RNeasy column clean-up (Qiagen, Valencia, CA, USA). Samples were stored at −80°C in 1 mM sodium citrate, pH 6.4 (Life Technologies, Grand Island, NY, USA) prior to cDNA synthesis. mRNA concentrations were measured using spectrophotometry and diluted to the same concentration for all samples. cDNA synthesis was preformed with MultiScribe™ MuLV reverse-transcriptase following the protocol for the high capacity cDNA synthesis kit (Life Technologies). cDNA was stored at −20°C. cDNA synthesis reaction was preformed in triplicate for vHC samples. Assays were performed using the TaqMan chemistry and off the shelf assays from Life Technologies. Assay IDs were: Gapdh-Mm99999915_g1 and cFos-Mm00487425_m1. Samples were run in triplicate on a Life Technologies 7900HT real-time PCR machine with a 20 μl reaction volume. Samples were compared using the ΔΔ*C*_T_ method of relative quantification. GAPDH was used to normalize between biological replicates.

### Statistics

All statistical analyses were accomplished using Prism 5 (GraphPad Software, La Jolla, CA, USA). Two-tailed *t*-tests and one-way or two-way ANOVAs were undertaken where appropriate, and Tukey *post hoc* tests were used to probe interactions. In all cases, results were considered statistically significant at the *P* = 0.05 level.

## Results

### CORT treatment does not affect body weight

After 4 weeks of treatment, no statistically significant difference was observed in weight gained between chronic CORT-treated animals and vehicle-treated animals (data not shown; *F*_1,37_ = 0.7057, *P* = 0.4063), replicating results from our previous work in this model (23, 25).

### CORT treatment results in altered diurnal patterns of plasma CORT

**Figure 1:**
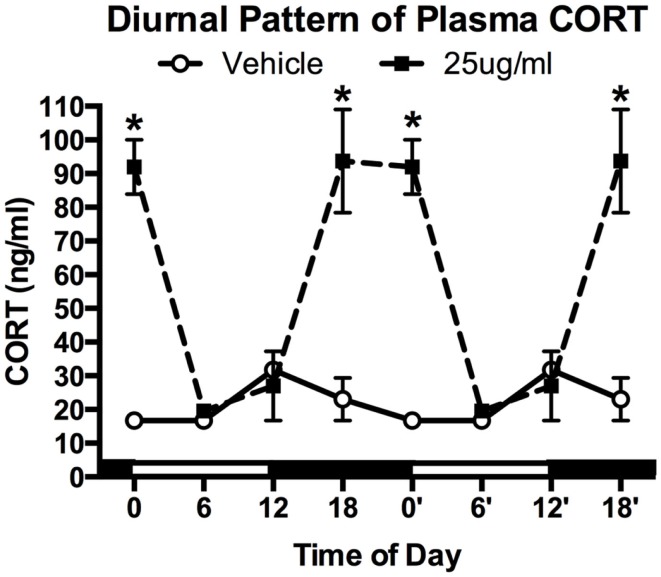
**Chronic low-dose CORT treatment in the drinking water maintains rhythmic, yet slightly higher, levels of plasma CORT**. Treatment with CORT (25 μg/ml) in the drinking water resulted in rhythmic diurnal changes in plasma CORT that reached a peak during the dark phase (ZT12–ZT0), but reached the same nadir levels as vehicle-treated mice (ZT6). Data from 0′ to 18′ are re-plotted from 0 to 18 to aid in visualization of the rhythm. Asterisks indicate statistically significant effects at the *P* < 0.05 level.

After 4 weeks of treatment, mice (*N* = 4–5/group) were killed at one of four time points: lights ON (ZT0), mid-light (ZT6), lights OFF (ZT12), and mid-night (ZT18). A significant main effect was detected for both treatment (two-way ANOVA; *F*_1,25_ = 44.29, *P* < 0.0001) and time (*F*_3,25_ = 13.39, *P* < 0.0001), as well as a significant interaction (*F*_3,25_ = 16.75, *P* < 0.0001). Tukey *post hoc* analysis showed that chronic CORT resulted in higher plasma CORT levels at both ZT18 and ZT0 (both *P* < 0.001), but nadir levels were statistically indistinguishable from vehicle-treated mice at ZT6 and ZT12 (both *P* > 0.05; Figure 1).

### CORT treatment results in a blunting of the endogenous stress response

**Figure 2:**
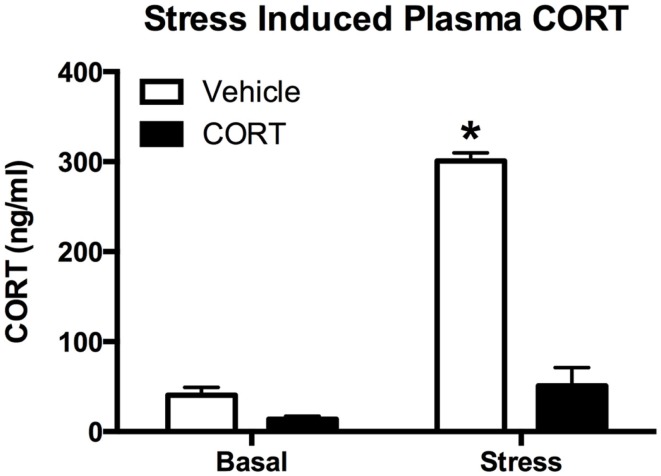
**Chronic low-dose CORT treatment blocks the normal endocrine response to stress**. Treatment with CORT (25 μg/ml) in the drinking water for 4 weeks resulted in reduced CORT secretion following a 10-min forced-swim stress at ZT6, with no effect on basal plasma CORT levels. Asterisk indicates statistically significant difference at the *P* < 0.05 level.

To determine how chronic CORT affects the peripheral (endocrine) stress response, we exposed CORT-treated mice to an acute FST stress. Given that ZT6 basal CORT levels were not different between vehicle- and chronic CORT-treated mice, we tested the endogenous hormonal stress response 10 min after a 10-min FST (*N* = 4–5/group). We found main effects of both CORT treatment (two-way ANOVA, *F*_1,15_ = 161.6, *P* < 0.0001) and stress (*F*_1,15_ = 187.7, *P* < 0.0001), and a significant interaction (*F*_1,15_ = 106.0, *P* < 0.0001). Tukey *post hoc* analysis showed that FST resulted in increased CORT only in vehicle-treated mice (*P* < 0.05), while stressed chronic CORT mice showed no increase in plasma CORT over basal levels (*P* > 0.05). There were no differences in non-stressed levels of plasma CORT between vehicle- and chronic CORT-treated mice (Figure 2). In a subset of only FST mice (*N* = 4–5/group), we also measured plasma ACTH at the same time point as CORT (Figure S1 in Supplementary Material), showing that CORT treatment blocked the normal increase in plasma ACTH following FST (*t*-test, *t* = 5.257, *P* = 0.0012). This suggests the endogenous hormonal stress response is impaired in CORT-treated mice.

### Chronic CORT treatment reduces corticotropin-releasing hormone mRNA in the paraventricular nucleus, but not the amygdala

**Figure 3:**
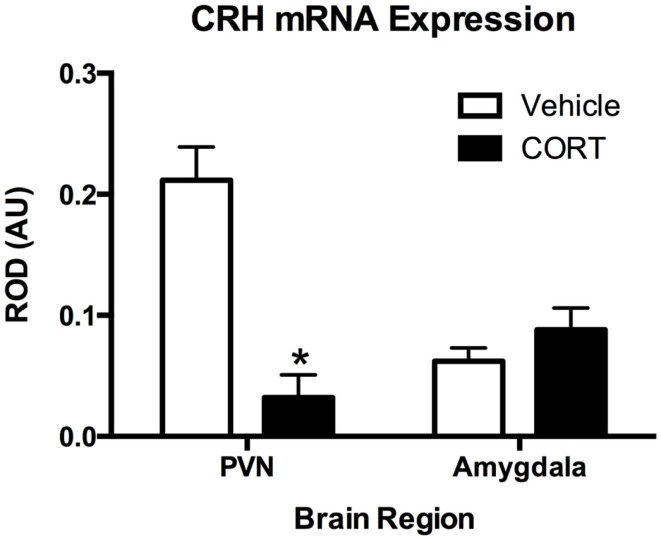
**Chronic low-dose CORT treatment reduced CRH mRNA expression in the PVN but does not affect expression in the amygdala**. *In situ* hybridization showed that treatment with CORT (25 μg/ml) in the drinking water for 4 weeks resulted in a statistically significant decrease in CRH mRNA expression in the PVN, but not in the amygdala. Asterisk indicates statistically significant difference at the *P* < 0.05 level.

To determine the effects of chronic CORT on central HPA function, using *in situ* hybridization, we assayed CRH mRNA levels following vehicle or CORT treatment (*N* = 4/group) within the PVN of the hypothalamus, a key central node in the HPA axis, and the amygdala (Figure 3). We found a main effect of CORT treatment and brain region (two-way ANOVA, *F*_1,12_ = 15.15., *P* = 0.002; *F*_1,12_ = 5.602, *P* = 0.0356), and a significant interaction (*F*_1,12_ = 27.21, *P* = 0.0002). Tukey *post hoc* analysis demonstrates that CRH mRNA levels are reduced in the PVN following 4 weeks of CORT treatment (*P* < 0.05), while CRH mRNA in the amygdala remained unaffected, compared to controls.

### Stress results in exaggerated *c*-Fos mRNA expression in CORT-treated mice

**Figure 4:**
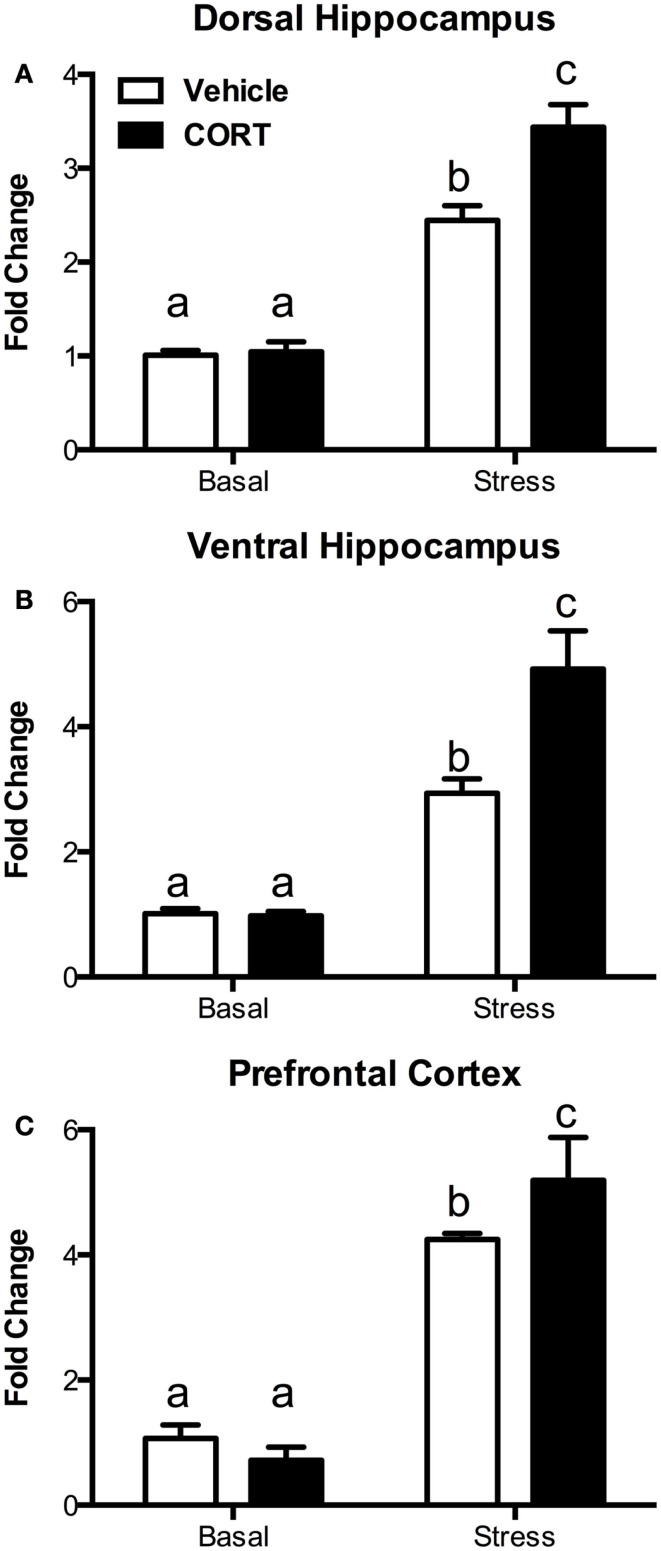
**Treatment with CORT in the drinking water results in exaggerated stress-induced *c-Fos* responses in the brain**. Following a 10-min forced-swim stress and a 10-min recovery period, CORT (25 μg/ml) drinking water-treated mice showed an exaggerated *c-Fos* mRNA response as measured by RT-PCR in **(A)** the dorsal hippocampus, **(B)** the ventral hippocampus, and **(C)** the prefrontal cortex. CORT treatment did not affect basal non-stressed levels of *c-Fos*. Columns that share the same letter are not statistically different from each other at the *P* < 0.05 level.

To determine if the blunting of the hormonal stress response was accompanied by changes in neural activation following the stress, we investigated changes in *c-Fos* mRNA using quantitative RT-PCR in the dorsal and ventral hippocampus (dHipp and vHipp), as well as in the prefrontal cortex (PFC) 10 min after the termination of a 10-min FST (Figure 4, *N* = 4–5/group).

In the dHipp (Figure 4A), we found significant main effects of both stress (two-way ANOVA, *F*_1,15_ = 149.4, *P* < 0.0001) and CORT treatment (*F*_1,15_ = 10.81, *P* = 0.005), as well as a significant interaction (*F*_1,15_ = 9.366, *P* = 0.0079). Tukey *post hoc* analysis showed that while stress increased dHipp *c-Fos* mRNA, the increase was greater in the chronic CORT-treated mice (*P* < 0.05), while there was no difference in non-stressed basal *c-Fos* expression.

Similarly, in the vHipp (Figure 4B), we found significant main effects of stress (*F*_1,14_ = 64.81, *P* < 0.0001) and chronic CORT treatment (*F*_1,14_ = 7.150, *P* = 0.0182), as well as a significant interaction (*F*_1,14_ = 7.655, *P* = 0.0151). *Post hoc* analysis indicated that, as in the dHipp, stress increased *c-Fos*, but this increase was greater in the chronic CORT mice (*P* < 0.05), without any differences in the non-stressed basal expression of *c-Fos*.

In the PFC (Figure 4C), while we found a significant main effect of stress (*F*_1,15_ = 373.2, *P* < 0.0001), we did not find a statistically significant main effect of CORT treatment (*F*_1,15_ = 2.282, *P* = 0.1516). However, there was a significant interaction (*F*_1,15_ = 10.75, *P* = 0.0051), with Tukey *post hoc* tests revealing a similar pattern where CORT-treated mice show enhanced *c-Fos* expression in the PFC following stress compared to vehicle-treated mice (*P* < 0.05), with no effect on non-stressed basal *c-Fos* expression.

Thus, in all instances, while chronic CORT does not affect basal non-stressed *c-Fos* expression, it does result in a statistically significant increase in *c-Fos* expression following FST in the dHipp, vHipp, and PFC.

### Chronic CORT treatment results in altered behavioral responses following stress

**Figure 5:**
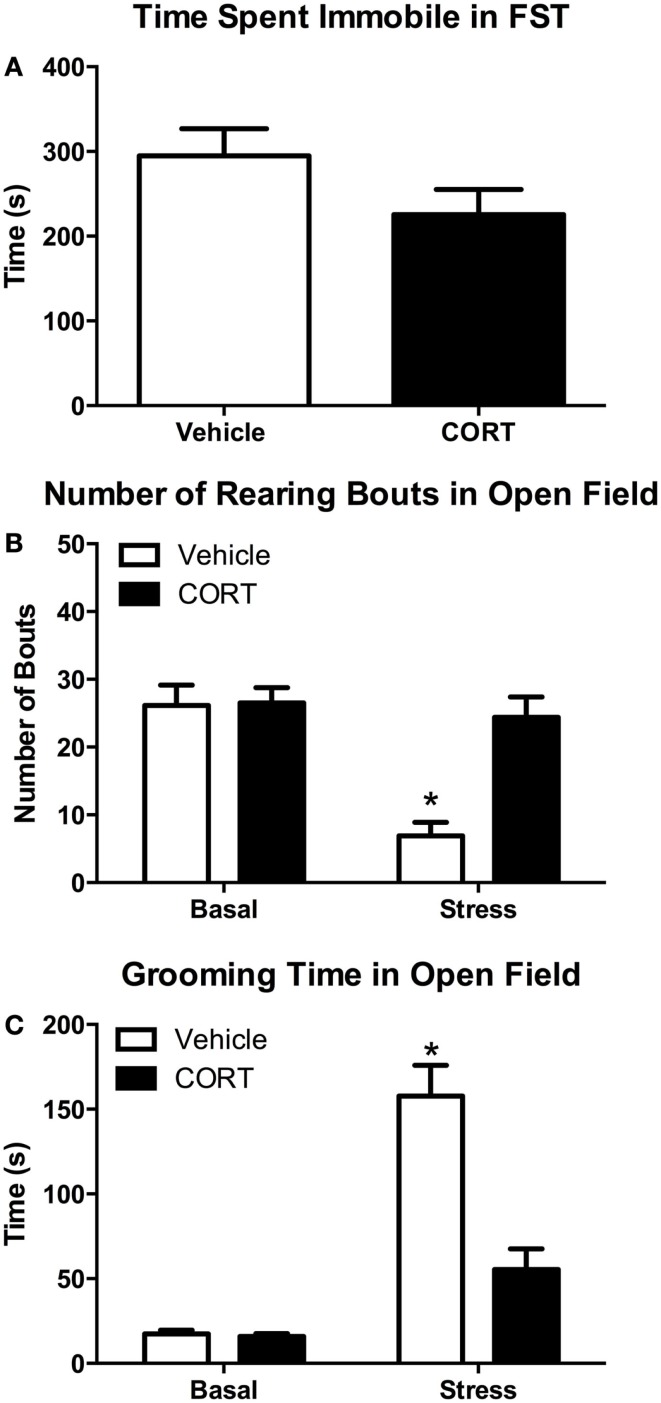
**Chronic low-dose CORT does not affect behavior in the FST, but blocks normal stress-induced anxiety behaviors in a novel environment after the FST**. Four weeks of CORT (25 μg/ml) in the drinking water does not alter time spent immobile in the FST **(A)**. Following the FST, vehicle-treated mice show subsequent anxiety-like behavior in an open field as measured by decreased rearing **(B)** and increased grooming **(C)**, while CORT-treated mice are statistically indistinguishable from controls, suggesting they do not show normal stress-induced anxiety-like behavior. Asterisks indicate statistically significant differences at the *P* < 0.05 level.

Given the altered hormonal and gene expression responses following acute FST stress in CORT-treated mice, we next wanted to determine if prior CORT treatment causes changes in acute stress-induced behavior. To these ends, we investigated stress-induced changes in swim behaviors in the FST, as well as rearing and grooming behavior in a novel open-field environment in vehicle- or CORT-treated mice, with or without stress (*N* = 8/group/treatment; Figure 5).

#### Behavior in the FST

Struggling in the FST was scored to determine if there was any effect of chronic low-dose CORT treatment on activity in the FST that may contribute to altered responses in rearing or grooming. We found that 4 weeks of chronic CORT did not affect time struggling or time spent immobile in the FST (Figure 5A; two-tailed *t*-test, *t* = 1.594, *P* = 0.13). We also found no effect of low-dose CORT on latency to immobility (data not shown; vehicle 58.63 ± 4.769 s vs. CORT 51.25 ± 2.782 s; two-tailed *t*-test, *t* = 1.336, *P* = 0.203).

#### Rearing behavior

One episode of forced-swim stress resulted in statistically significant differences in the frequency of rearing behavior in the OFT (Figure 5B). While there was a main effect of FST stress (*F*_1,28_ = 16.64, *P* = 0.0003) and CORT treatment (*F*_1,28_ = 11.64, *P* = 0.0020) on rearing frequency, there was also a statistically significant interaction between stress and CORT treatment (*F*_1,28_ = 10.68, *P* = 0.0029). *Post hoc* analyses revealed that VEH mice showed a significant decrease in grooming (*P* < 0.0003). However, no statistically significant difference in rearing frequency was observed between stress/CORT animals and non-stress/VEH animals (*P* = 0.9645) or non-stress/CORT animals (*P* = 0.9392), suggesting chronic CORT blocked the normal stress-induced decreases in rearing behavior.

#### Grooming behavior

Similar to the effects on rearing behavior, FST and CORT treatment resulted in main effects on grooming duration (Figure 5C; *F*_1,27_ = 62.36, *P* < 0.0001 and *F*_1,27_ = 20.83, *P* < 0.0001, respectively). However, there was also a statistically significant interaction between stress and CORT treatment (*F*_1,27_ = 19.60, *P* < 0.0001). *Post hoc* analyses revealed that FST increased grooming duration in VEH-treated mice (*P* < 0.0001). However, no statistically significant difference in grooming duration was observed between stress/CORT mice and non-stress/VEH mice (*P* = 0.1191), or non-stress/CORT mice (*P* = 0.0832), further supporting the notion that CORT induced disruption of the HPA blocks normal behavioral responses to acute stress.

## Discussion

The stress response is an essential brain–body response that protects homeostasis and ensures survival in the face of threatening environmental stimuli (12, 28–30). The coordinated response following stress allows for mediators of allostasis to mobilize and allow an organism to adapt to a temporary environmental shift. As such, these responses can be considered protective, and in fact, healthy allostatic responses are a sign of resilience (31–33). However, if allostatic mediators are over active, improperly regulated, poorly terminated, or engaged inappropriately, allostatic load can increase. Eventually this results in allostatic overload, which, rather than protecting the organism, leads to a series of cascading failures in multiple physiological systems. Thus, the systems that usually impart resilience instead result in increased vulnerability (12, 31, 32, 34). Given the central role of the HPA axis stress response in allostatic responses, we probed how our model of disrupted HPA function might lead to altered neural and behavioral outputs, and perhaps contribute to negative mental and physical health outcomes.

Disruption of normal HPA function is a hallmark of a varied set of physical and neuropsychiatric diseases, such as obesity, depression, and anxiety (21, 22, 35). For example, PTSD is associated with dysregulated HPA function. Even though some reports suggest a hyperactive HPA is related to development of PTSD (36, 37), while others propose that HPA hypoactivity may be a key factor (38–40), the overarching theme remains: that in PTSD the HPA is disrupted and does not respond normally to stressors. Whether these changes in HPA activity are a cause or consequence of such disease states is unclear, though it is feasible that disrupting the normal responses to environmental stress could predispose individuals to negative health outcomes. In the present study, we explored how disruption of the HPA via a non-invasive drinking water treatment could change the normal neuroendocrine stress response, and how this change would affect both neural and behavioral outputs following exposure to an acute stress.

The present study used a non-invasive manipulation of the HPA that maintains near normal diurnal changes in plasma CORT, but interferes with the neuroendocrine stress response. This model is a variant of more classic approaches used in adrenalectomized rats (41, 42). In our model, mice treated with a low-dose (25 μg/ml) of CORT in the drinking water for 4 weeks show normal growth curves (23) and show a diurnal rhythm in plasma CORT that is somewhat higher, and peaks slightly later, than CORT in un-manipulated mice (Figure 1). In the present study, we further report that this model results in the shutdown of the normal endocrine response to forced-swim stress, with no increase in plasma CORT observed following the stress (Figure 2). This is likely due to both peripheral, as we have previously demonstrated that it also leads to atrophy of the adrenal gland (23, 25), as well as central effects, since we observed a decrease in PVN CRH mRNA (Figure 3). Given this altered plasma CORT response, the purpose of the second part of the study was to determine if disruption of the HPA response would result in differential neural and behavioral responses to acute stress.

Acute stress results in a sequence of neural and behavioral responses, and perturbations of these normal responses may indicate underlying faults in the neural and endocrine systems regulating stress reactivity. Following stress, brain *c-Fos* expression is increased in the PFC and HIPP, as well as in a number of other brain areas (43–45), suggesting increased neuronal activity. However, the consequences of altered HPA function for stress-induced *c-Fos* expression are unclear. Some reports indicate that ADX has no effect on acute stress-induced *c-Fos* mRNA (46), while others describe increases in stress-induced *c-Fos* expression following ADX (47, 48). Given these somewhat conflicting reports, we chose to explore both neural and behavioral responses in a model of HPA dysfunction. In our model, the normally observed increase in plasma CORT following stress is abolished (Figure 2), and levels of PVN CRH mRNA are reduced (Figure 3). This led us to posit that normal neural responses to stress would also be blunted, given that the primary neuroendocrine output of the stress response is greatly reduced. However, our hypothesis was proven incorrect, and instead we observed increased neural responses in the brain regions we explored (Figure 4).

Following stress, many brain regions in addition to the PFC and HIPP show increased activity (2, 3, 46, 49), forming an almost brain-wide network from the brain stem through to the forebrain. For instance, in response to a novel environmental stressor, noradrenergic neurons of the locus ceruleus (LC) rapidly increase firing rate. These cells are known to project to forebrain regions such as the PFC and HIPP where they play a largely excitatory role (50, 51). Many of these same areas are also intimately involved in the negative feedback aspect of the stress response, with activation of the GR serving to reduce their activity. For example, in ADX rats, a single bolus injection of CORT leads to a decrease in *c-Fos* expression in the dorsal HIPP within 45 min (52). In the present study, we determined that even though our model results in decreased plasma CORT in response to stress (Figure 2), neural activation (as measured by *c-Fos* mRNA) is significantly increased in the PFC, as well as the dorsal and ventral hippocampus (dHipp and vHipp; Figure 4). These novel and unexpected findings have led us to hypothesize that the lack of a normal endogenous CORT response in exogenous CORT-treated animals may deprive these circuits of a key negative feedback signal. Such attenuation of neural activity by glucocorticoids has been observed in LC noradrenergic neurons, where activation of the GR in LC neurons decreases tyrosine hydroxylase mRNA and presumably reduces neuronal activity (53). This leads to a possible mechanism that explains the observed increased *c-Fos* expression in the mPFC and hippocampus in CORT-treated animals: i.e., it is possible that without the inhibitory drive of CORT to the LC following a stressor, mPFC and hippocampal activity is increased above “normal” levels via overstimulation by noradrenergic LC afferents to these regions. Such an outcome could be tested in future studies by specifically manipulating different components of this circuit, with or without chronic CORT treatment.

Given the obvious differences in both hormonal and neural responses to stress in CORT-treated mice, and to clarify the functional consequences of these altered responses, we next wanted to explore changes in behavioral outputs following stress. It is well documented that acute stress can lead to increased anxiety-like behaviors in rodents on a variety of measures, including the elevated plus maze, open field, and social avoidance (54–58). These alterations in behavior are mediated by both neural and hormonal changes during and following the stressor. To determine the behavioral consequences of a blunted hormonal stress response, but exaggerated neural stress response, we analyzed behavioral changes in the novel environment of an open field following a forced-swim stress (Figure 5). Rearing in a novel open field can be considered a form of exploratory behavior (59), associated with positive affective states and thus indicative of low anxiety (60). Our results indicate that in response to an acute stressor, animals chronically treated with CORT fail to display the normally observed decrease in rearing behavior. From this, we conclude that CORT treatment reduces anxiety responses normally elicited by a novel acute stress. Similarly, self-grooming behavior in rodents has been associated with high levels of anxiety (61, 62). In the present study, we observe lower duration of grooming in CORT-treated animals compared to those treated with vehicle in response to an acute stress. Chronic CORT-treated animals resemble non-stressed animals in that grooming duration does not significantly increase following stress. Thus, we conclude that the failure to increase grooming duration following acute stress in CORT-treated compared to vehicle-treated mice again suggests a lower anxiety state in CORT-treated mice. It is important to note that we did not find behavioral differences between the vehicle and CORT groups in the FST (Figure 5A), suggesting the neural and behavioral responses after FST are not simply due to differences in amount of exercise or stress coping strategies. Gourley et al. noted a slight increase in immobility in the tail suspension test using a similar model (63), but it is important to note that their results used CORT hemisuccinate (that may alter bioavailability), and a 3-day “washout” period, making direct comparison difficult as our mice were still being treated with CORT at time of testing. We conclude that disrupting the HPA axis using this model of chronic CORT in the drinking water blocks normal behavioral responses to acute stress.

The behavior of CORT-treated mice following acute stress is statistically indistinguishable from non-stressed mice with respect to grooming and rearing behaviors in the open field (Figure 5). On the other hand, activity in the PFC and HIPP clearly shows significant increase in *c-Fos* expression, to a level greater than both stressed vehicle mice as well as non-stressed vehicle or CORT mice (Figure 4). Thus, there appears to be a mismatch between the neural and behavioral responses to stress in CORT-treated mice that have a dysregulated HPA. It is intriguing to consider the ramifications of such “inappropriate” behavioral and neural responses following stress. One interpretation could be that the inability of CORT-treated mice to increase plasma CORT levels following a stressor results in a protective phenotype that does not show increases in anxiety-like behavior. However, such resilience should not necessarily be construed as a beneficial consequence to the organism, as blunted reactions to stress could be maladaptive. Acute environmental stressors produce necessary neurobehavioral responses that improve chances for survival in challenging environments. For instance, in many cases, memory and learning processes are increased by acute stress (64–66), with the presumed benefit of allowing for future avoidance of the stressor or context in which the stressor occurred (67). Restated, behavioral, and physiological phenotypes normally expressed following exposure to acute stress are thought to be adaptive. Thus, reduction of these behaviors by HPA dysregulation observed here could be described as maladaptive. This suggests it is equally plausible to consider the alternative interpretation: that the lack of a behavioral response following a stressor, in association with increased activity in the PFC and HIPP, may have negative consequences because the appropriate allostatic mediators are not being adequately mobilized, and/or their termination is not being efficiently regulated. Perhaps in the short term, such responses have little negative impact, but repeated stress exposure without the normal hormonal, neural, or behavioral responses may contribute to increased allostatic load and eventual long-term negative outcomes.

The results of our study suggest the low-dose CORT model (without ADX) may be useful in the investigation of disorders in which a dysregulation of the HPA axis, or other aspects of the stress response, are observed, such as PTSD, depression, and anxiety disorders. As we have shown that many gross markers of adrenal status recover after removal of the CORT treatment (25), the reversibility of this model could provide insight into long-term effects of short-term HPA dysregulation. By devising models that can be dissected both anatomically and temporally, we may gain increased understanding of the neural and behavioral underpinnings of complex neuropsychiatric disorders.

## Conflict of Interest Statement

The authors declare that the research was conducted in the absence of any commercial or financial relationships that could be construed as a potential conflict of interest.

## Supplementary Material

The Supplementary Material for this article can be found online at http://www.frontiersin.org/Journal/10.3389/fpsyt.2015.00031/abstract
