# Supplementary material for: Dysregulated Hypothalamic–Pituitary–Adrenal Axis Function Contributes to Altered Endocrine and Neurobehavioral Responses to Acute Stress
Source: Front Psychiatry. 2015 Mar 13;6:31. doi: 10.3389/fpsyt.2015.00031 (PMC4358064; doi:10.3389/fpsyt.2015.00031)
Supplement: Supplementary file 1 [file Image_1.PDF]

SUPPLEMENTAL FIGURE 1

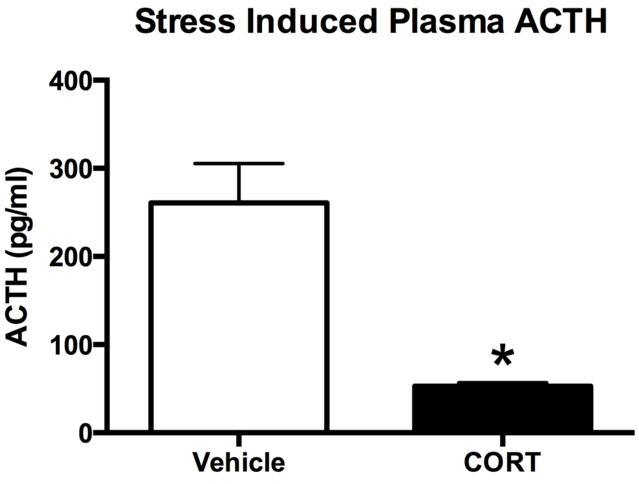

Supplemental Figure 1.

Chronic CORT treatment blunts the stress-induced plasma ACTH response. Plasma ACTH levels were assessed only in stressed Vehicle or CORT treated mice (N=4-5/group). The results show that 28d of chronic low dose (25μg/ml) CORT treatment blunts the normal stress induced increase in plasma ACTH. Asterisk indicates a statistically significant difference. Two-tailed t-test, t=5.257, P = 0.0012.
